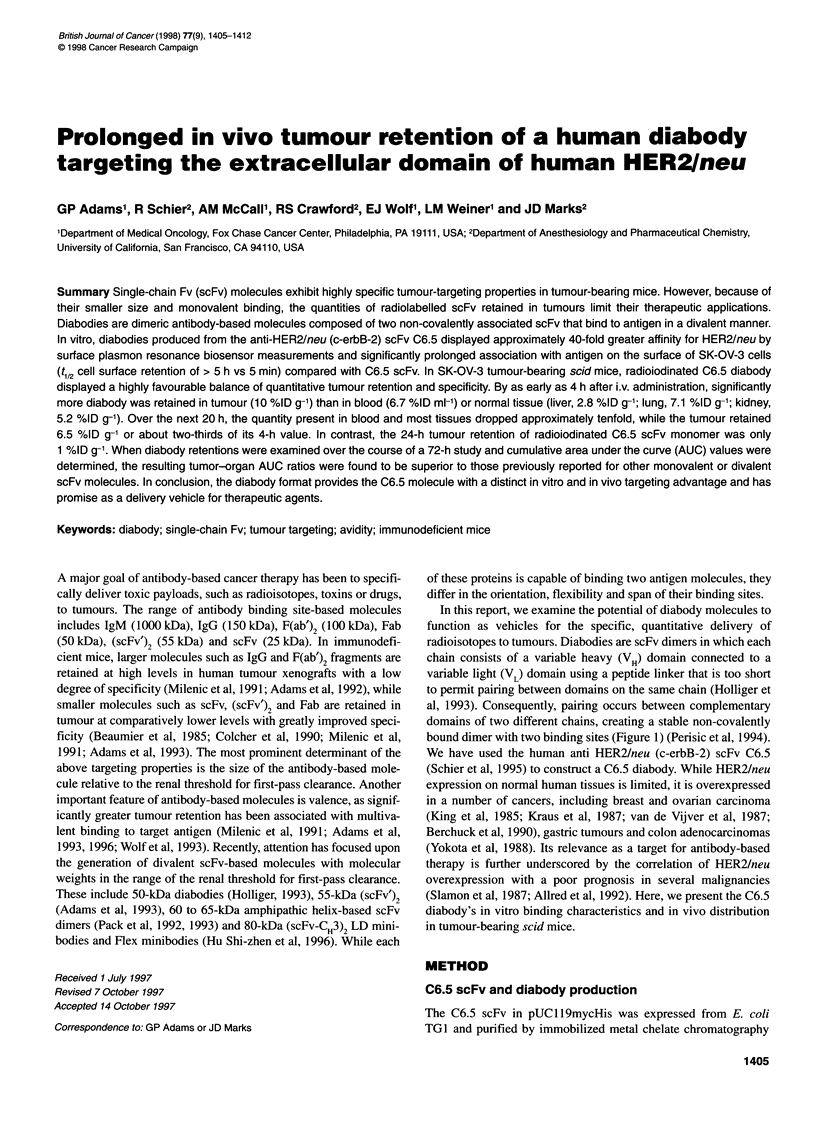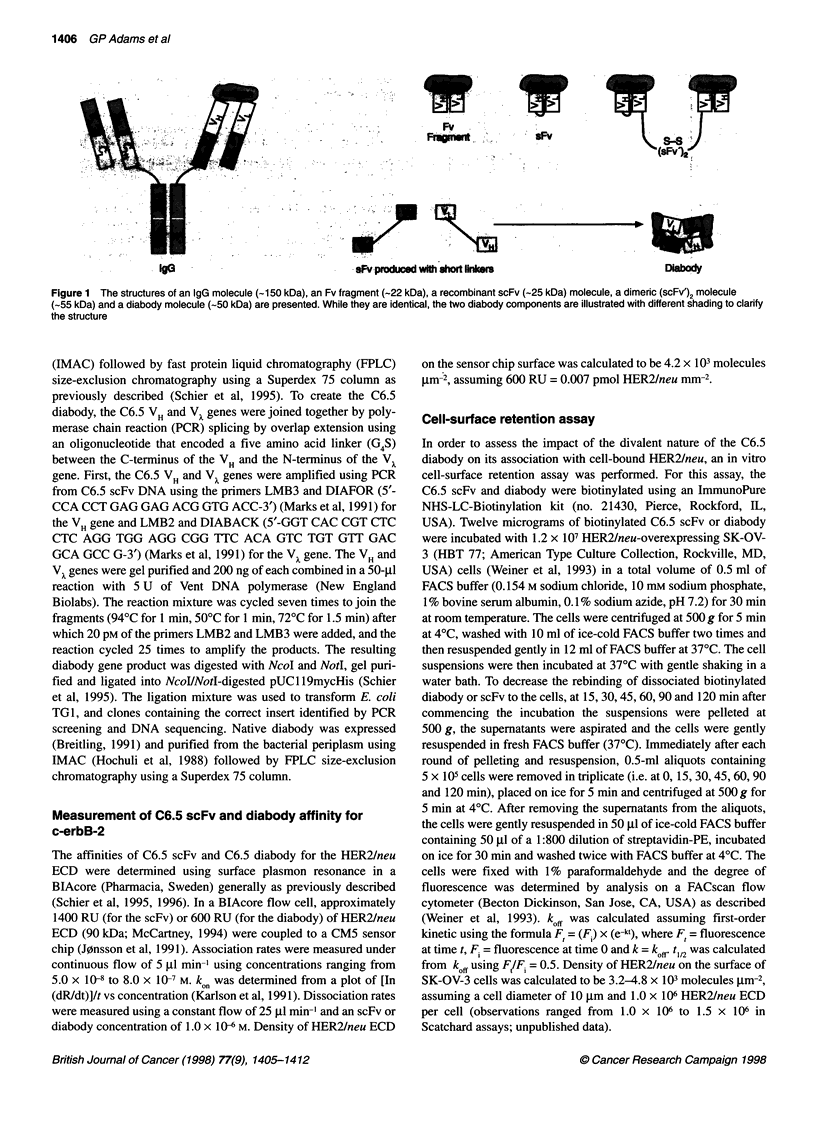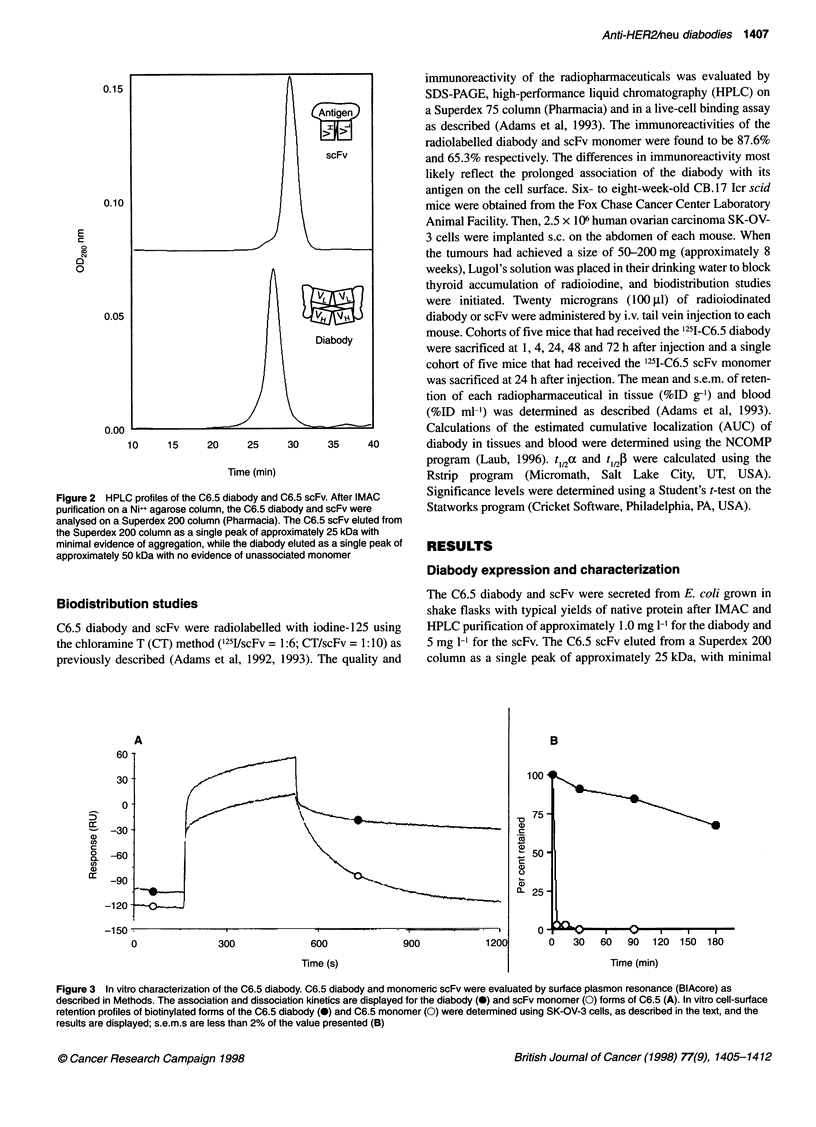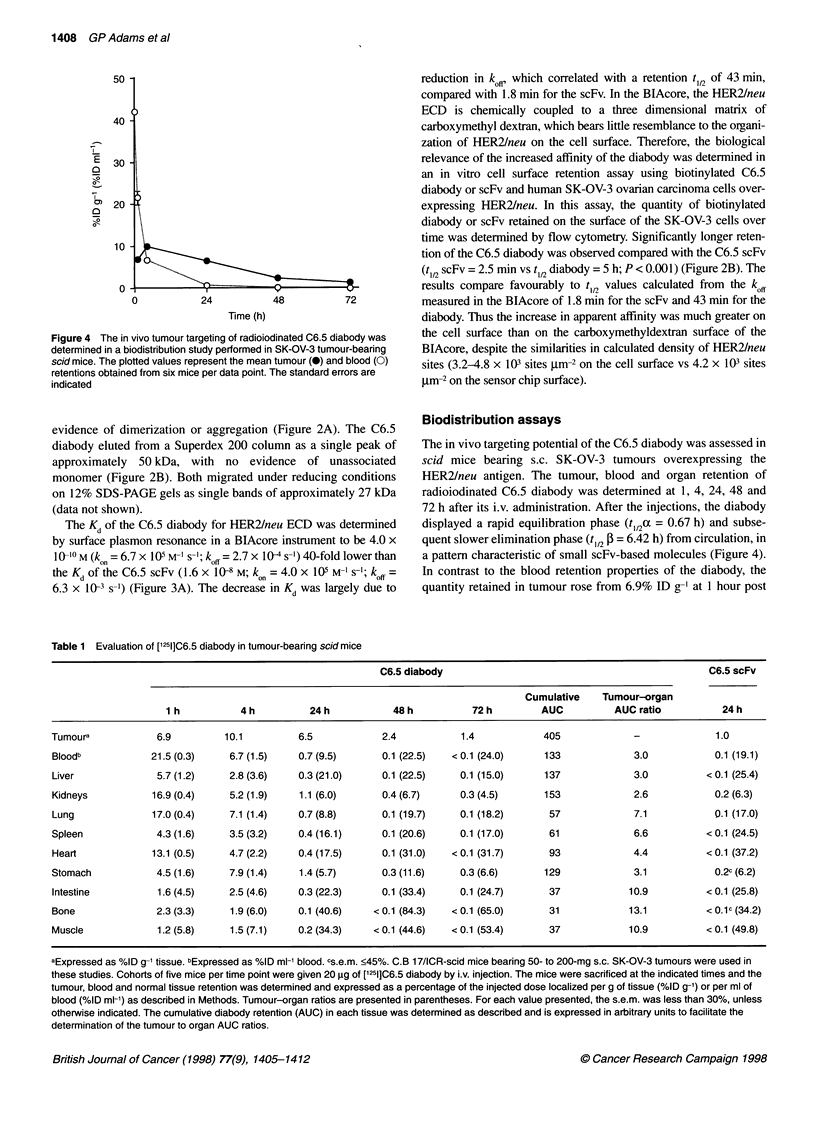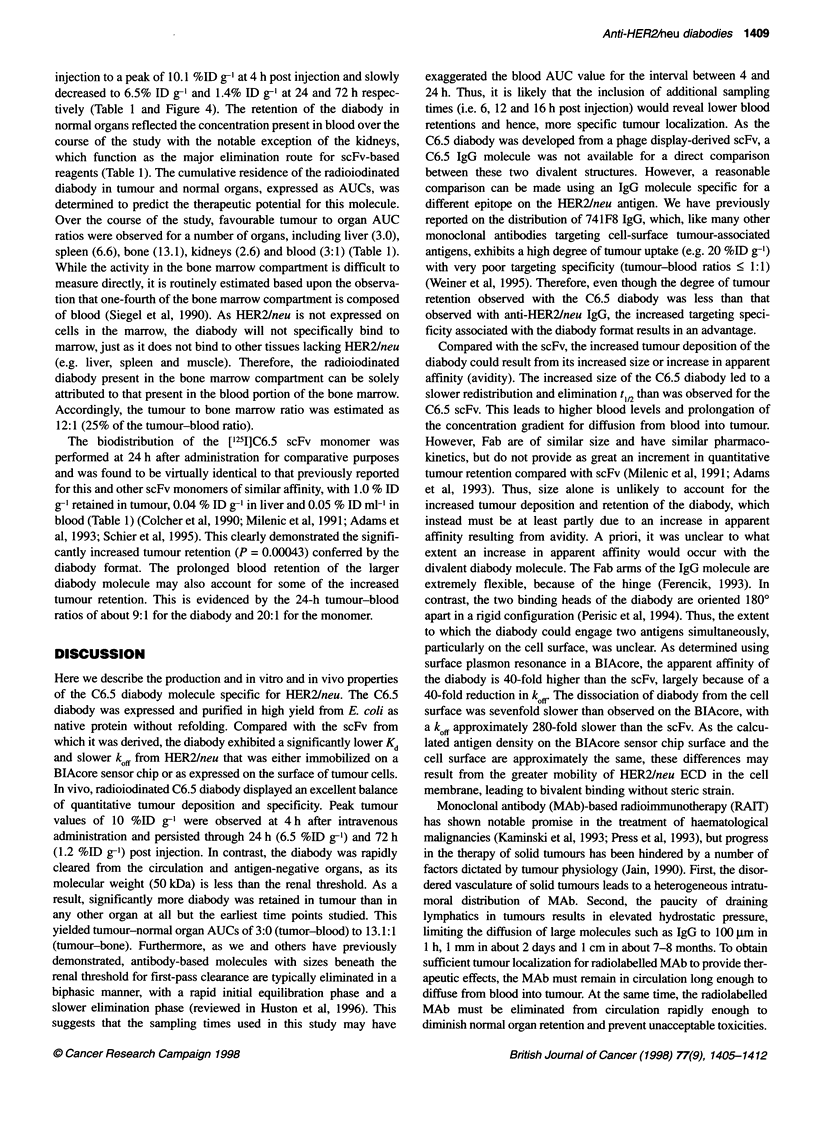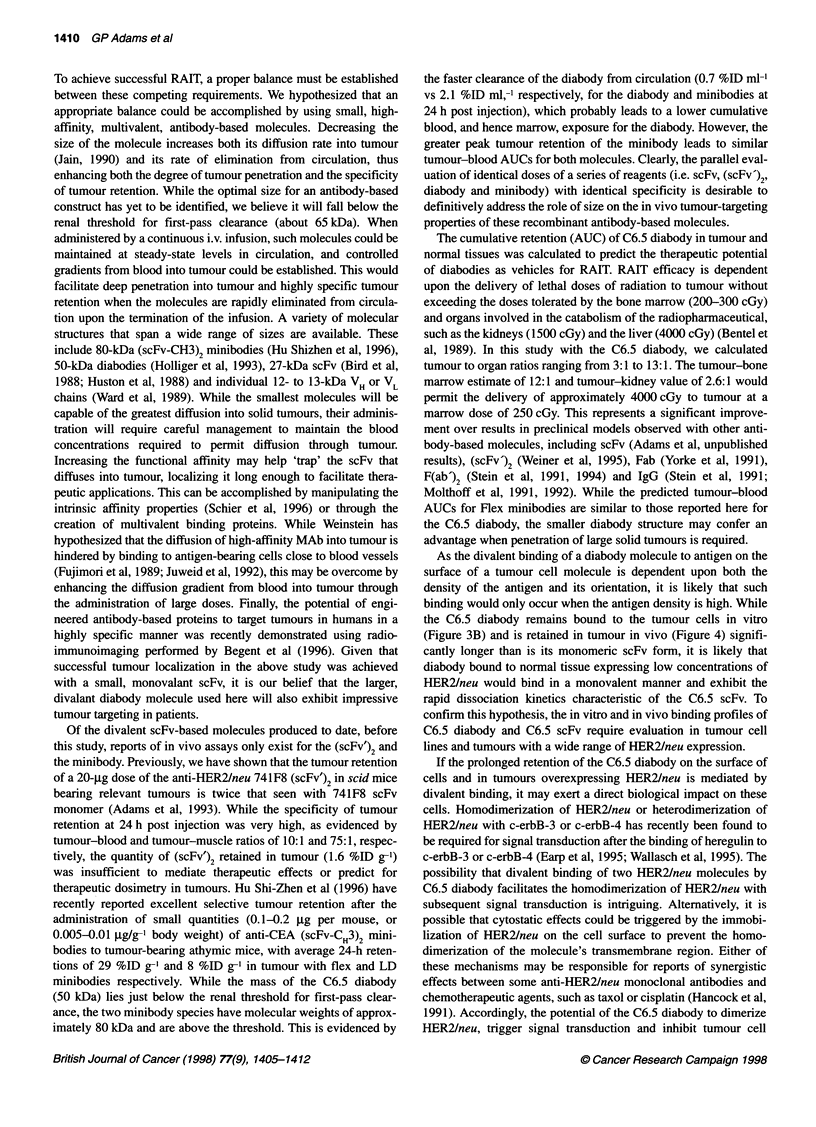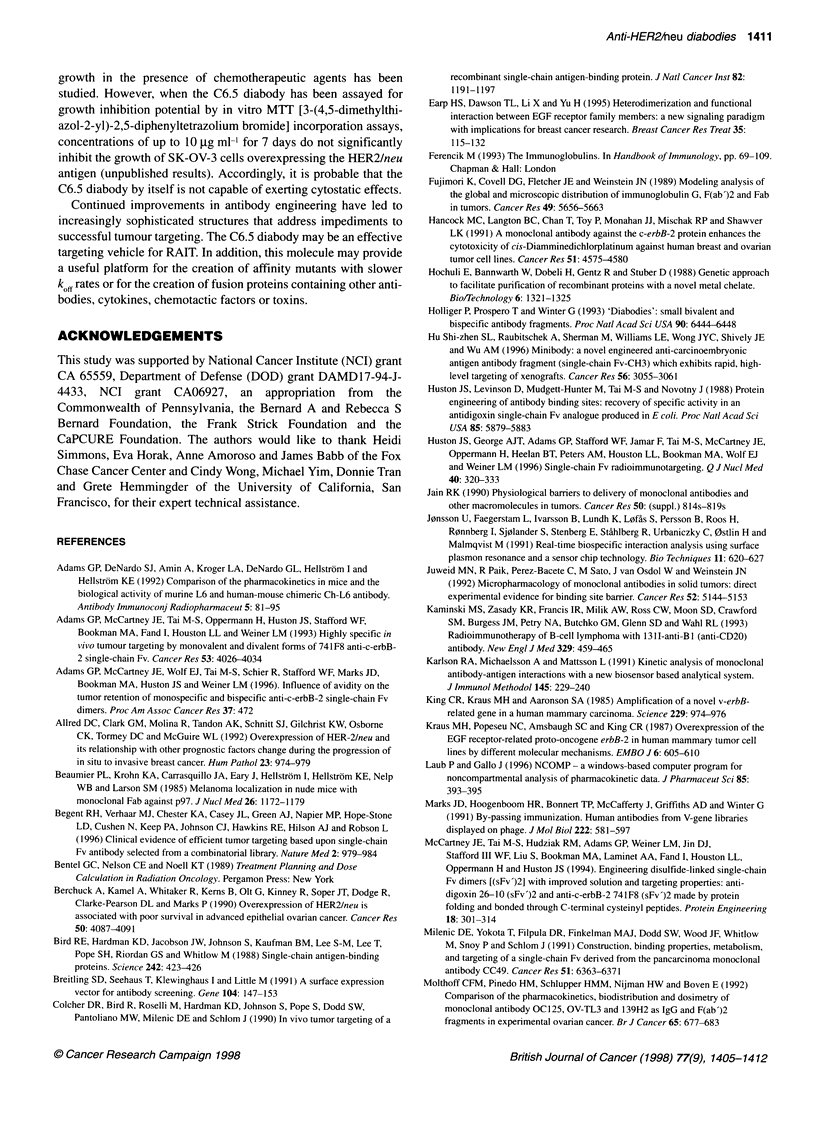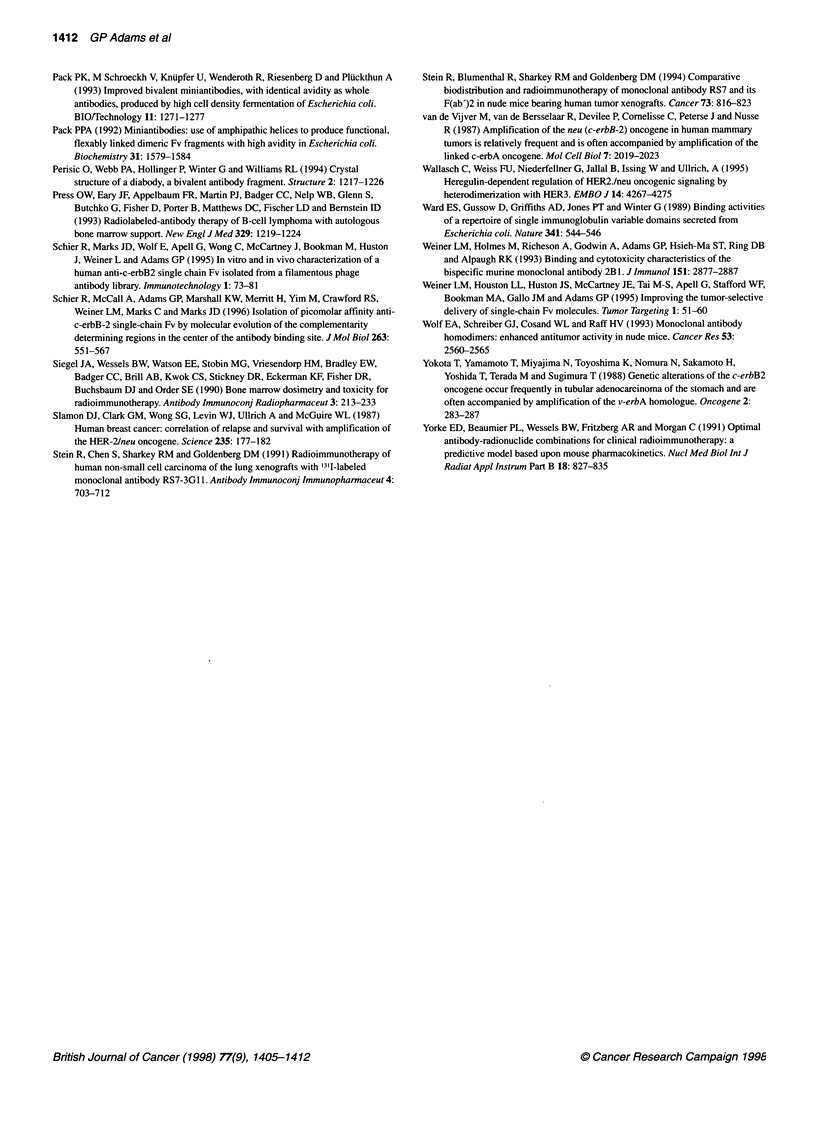# Prolonged in vivo tumour retention of a human diabody targeting the extracellular domain of human HER2/neu.

**DOI:** 10.1038/bjc.1998.233

**Published:** 1998-05

**Authors:** G. P. Adams, R. Schier, A. M. McCall, R. S. Crawford, E. J. Wolf, L. M. Weiner, J. D. Marks

**Affiliations:** Department of Medical Oncology, Fox Chase Cancer Center, Philadelphia, PA 19111, USA.

## Abstract

Single-chain Fv (scFv) molecules exhibit highly specific tumour-targeting properties in tumour-bearing mice. However, because of their smaller size and monovalent binding, the quantities of radiolabelled scFv retained in tumours limit their therapeutic applications. Diabodies are dimeric antibody-based molecules composed of two non-covalently associated scFv that bind to antigen in a divalent manner. In vitro, diabodies produced from the anti-HER2/neu (c-erbB-2) scFv C6.5 displayed approximately 40-fold greater affinity for HER2/neu by surface plasmon resonance biosensor measurements and significantly prolonged association with antigen on the surface of SK-OV-3 cells (t1/2 cell surface retention of > 5 h vs 5 min) compared with C6.5 scFv. In SK-OV-3 tumour-bearing scid mice, radioiodinated C6.5 diabody displayed a highly favourable balance of quantitative tumour retention and specificity. By as early as 4 h after i.v. administration, significantly more diabody was retained in tumour (10 %ID g(-1)) than in blood (6.7 %ID ml(-1)) or normal tissue (liver, 2.8 %ID g(-1); lung, 7.1 %ID g(-1); kidney, 5.2 %ID g(-1)). Over the next 20 h, the quantity present in blood and most tissues dropped approximately tenfold, while the tumour retained 6.5 %ID g(-1) or about two-thirds of its 4-h value. In contrast, the 24-h tumour retention of radioiodinated C6.5 scFv monomer was only 1 %ID g(-1). When diabody retentions were examined over the course of a 72-h study and cumulative area under the curve (AUC) values were determined, the resulting tumor-organ AUC ratios were found to be superior to those previously reported for other monovalent or divalent scFv molecules. In conclusion, the diabody format provides the C6.5 molecule with a distinct in vitro and in vivo targeting advantage and has promise as a delivery vehicle for therapeutic agents.


British Journal of Cancer (1998) 77(9), 1405–1412
© 1998 Cancer Research Campaign

### Prolonged in vivo tumour retention of a human diabody targeting the extracellular domain of human HER2/*neu*

**GP Adams[1], R Schier[2], AM McCall[1], RS Crawford[2], EJ Wolf[1], LM Weiner[1] and JD Marks[2]**

[1]Department of Medical Oncology, Fox Chase Cancer Center, Philadelphia, PA 19111, USA; [2]Department of Anesthesiology and Pharmaceutical Chemistry, University of California, San Francisco, CA 94110, USA

**Summary** Single-chain Fv (scFv) molecules exhibit highly specific tumour-targeting properties in tumour-bearing mice. However, because of their smaller size and monovalent binding, the quantities of radiolabelled scFv retained in tumours limit their therapeutic applications. Diabodies are dimeric antibody-based molecules composed of two non-covalently associated scFv that bind to antigen in a divalent manner. In vitro, diabodies produced from the anti-HER2/*neu* (c-erbB-2) scFv C6.5 displayed approximately 40-fold greater affinity for HER2/*neu* by surface plasmon resonance biosensor measurements and significantly prolonged association with antigen on the surface of SK-OV-3 cells ($t_{1/2}$ cell surface retention of > 5 h vs 5 min) compared with C6.5 scFv. In SK-OV-3 tumour-bearing *scid* mice, radioiodinated C6.5 diabody displayed a highly favourable balance of quantitative tumour retention and specificity. By as early as 4 h after i.v. administration, significantly more diabody was retained in tumour (10 %ID g$^{-1}$) than in blood (6.7 %ID ml$^{-1}$) or normal tissue (liver, 2.8 %ID g$^{-1}$; lung, 7.1 %ID g$^{-1}$; kidney, 5.2 %ID g$^{-1}$). Over the next 20 h, the quantity present in blood and most tissues dropped approximately tenfold, while the tumour retained 6.5 %ID g$^{-1}$ or about two-thirds of its 4-h value. In contrast, the 24-h tumour retention of radioiodinated C6.5 scFv monomer was only 1 %ID g$^{-1}$. When diabody retentions were examined over the course of a 72-h study and cumulative area under the curve (AUC) values were determined, the resulting tumor–organ AUC ratios were found to be superior to those previously reported for other monovalent or divalent scFv molecules. In conclusion, the diabody format provides the C6.5 molecule with a distinct in vitro and in vivo targeting advantage and has promise as a delivery vehicle for therapeutic agents.

**Keywords:** diabody; single-chain Fv; tumour targeting; avidity; immunodeficient mice

A major goal of antibody-based cancer therapy has been to specifically deliver toxic payloads, such as radioisotopes, toxins or drugs, to tumours. The range of antibody binding site-based molecules includes IgM (1000 kDa), IgG (150 kDa), F(ab′)$_2$ (100 kDa), Fab (50 kDa), (scFv′)$_2$ (55 kDa) and scFv (25 kDa). In immunodeficient mice, larger molecules such as IgG and F(ab′)$_2$ fragments are retained at high levels in human tumour xenografts with a low degree of specificity (Milenic et al, 1991; Adams et al, 1992), while smaller molecules such as scFv, (scFv′)$_2$ and Fab are retained in tumour at comparatively lower levels with greatly improved specificity (Beaumier et al, 1985; Colcher et al, 1990; Milenic et al, 1991; Adams et al, 1993). The most prominent determinant of the above targeting properties is the size of the antibody-based molecule relative to the renal threshold for first-pass clearance. Another important feature of antibody-based molecules is valence, as significantly greater tumour retention has been associated with multivalent binding to target antigen (Milenic et al, 1991; Adams et al, 1993, 1996; Wolf et al, 1993). Recently, attention has focused upon the generation of divalent scFv-based molecules with molecular weights in the range of the renal threshold for first-pass clearance. These include 50-kDa diabodies (Holliger, 1993), 55-kDa (scFv′)$_2$ (Adams et al, 1993), 60 to 65-kDa amphipathic helix-based scFv dimers (Pack et al, 1992, 1993) and 80-kDa (scFv-C$_H$3)$_2$ LD minibodies and Flex minibodies (Hu Shi-zhen et al, 1996). While each of these proteins is capable of binding two antigen molecules, they differ in the orientation, flexibility and span of their binding sites.

In this report, we examine the potential of diabody molecules to function as vehicles for the specific, quantitative delivery of radioisotopes to tumours. Diabodies are scFv dimers in which each chain consists of a variable heavy (V$_H$) domain connected to a variable light (V$_L$) domain using a peptide linker that is too short to permit pairing between domains on the same chain (Holliger et al, 1993). Consequently, pairing occurs between complementary domains of two different chains, creating a stable non-covalently bound dimer with two binding sites (Figure 1) (Perisic et al, 1994). We have used the human anti HER2/*neu* (c-erbB-2) scFv C6.5 (Schier et al, 1995) to construct a C6.5 diabody. While HER2/*neu* expression on normal human tissues is limited, it is overexpressed in a number of cancers, including breast and ovarian carcinoma (King et al, 1985; Kraus et al, 1987; van de Vijver et al, 1987; Berchuck et al, 1990), gastric tumours and colon adenocarcinomas (Yokota et al, 1988). Its relevance as a target for antibody-based therapy is further underscored by the correlation of HER2/*neu* overexpression with a poor prognosis in several malignancies (Slamon et al, 1987; Allred et al, 1992). Here, we present the C6.5 diabody's in vitro binding characteristics and in vivo distribution in tumour-bearing *scid* mice.

*Received 1 July 1997*
*Revised 7 October 1997*
*Accepted 14 October 1997*

*Correspondence to:* GP Adams or JD Marks

### METHOD

### C6.5 scFv and diabody production

The C6.5 scFv in pUC119mycHis was expressed from *E. coli* TG1 and purified by immobilized metal chelate chromatography

**Figure 1** The structures of an IgG molecule (~150 kDa), an Fv fragment (~22 kDa), a recombinant scFv (~25 kDa) molecule, a dimeric (scFv′)$_2$ molecule (~55 kDa) and a diabody molecule (~50 kDa) are presented. While they are identical, the two diabody components are illustrated with different shading to clarify the structure

(IMAC) followed by fast protein liquid chromatography (FPLC) size-exclusion chromatography using a Superdex 75 column as previously described (Schier et al, 1995). To create the C6.5 diabody, the C6.5 $V_H$ and $V_\lambda$ genes were joined together by polymerase chain reaction (PCR) splicing by overlap extension using an oligonucleotide that encoded a five amino acid linker ($G_4S$) between the C-terminus of the $V_H$ and the N-terminus of the $V_\lambda$ gene. First, the C6.5 $V_H$ and $V_\lambda$ genes were amplified using PCR from C6.5 scFv DNA using the primers LMB3 and DIAFOR (5′-CCA CCT GAG GAG ACG GTG ACC-3′) (Marks et al, 1991) for the $V_H$ gene and LMB2 and DIABACK (5′-GGT CAC CGT CTC CTC AGG TGG AGG CGG TTC ACA GTC TGT GTT GAC GCA GCC G-3′) (Marks et al, 1991) for the $V_\lambda$ gene. The $V_H$ and $V_\lambda$ genes were gel purified and 200 ng of each combined in a 50-µl reaction with 5 U of Vent DNA polymerase (New England Biolabs). The reaction mixture was cycled seven times to join the fragments (94°C for 1 min, 50°C for 1 min, 72°C for 1.5 min) after which 20 pM of the primers LMB2 and LMB3 were added, and the reaction cycled 25 times to amplify the products. The resulting diabody gene product was digested with *Nco*I and *Not*I, gel purified and ligated into *NcoI*/*Not*I-digested pUC119mycHis (Schier et al, 1995). The ligation mixture was used to transform *E. coli* TG1, and clones containing the correct insert identified by PCR screening and DNA sequencing. Native diabody was expressed (Breitling, 1991) and purified from the bacterial periplasm using IMAC (Hochuli et al, 1988) followed by FPLC size-exclusion chromatography using a Superdex 75 column.

### Measurement of C6.5 scFv and diabody affinity for c-erbB-2

The affinities of C6.5 scFv and C6.5 diabody for the HER2/*neu* ECD were determined using surface plasmon resonance in a BIAcore (Pharmacia, Sweden) generally as previously described (Schier et al, 1995, 1996). In a BIAcore flow cell, approximately 1400 RU (for the scFv) or 600 RU (for the diabody) of HER2/*neu* ECD (90 kDa; McCartney, 1994) were coupled to a CM5 sensor chip (Jønsson et al, 1991). Association rates were measured under continuous flow of 5 µl $min^{-1}$ using concentrations ranging from $5.0 \times 10^{-8}$ to $8.0 \times 10^{-7}$ M. $k_{on}$ was determined from a plot of [In (dR/dt)]/*t* vs concentration (Karlson et al, 1991). Dissociation rates were measured using a constant flow of 25 µl $min^{-1}$ and an scFv or diabody concentration of $1.0 \times 10^{-6}$ M. Density of HER2/*neu* ECD on the sensor chip surface was calculated to be $4.2 \times 10^3$ molecules $\mu m^{-2}$, assuming 600 RU = 0.007 pmol HER2/*neu* $mm^{-2}$.

### Cell-surface retention assay

In order to assess the impact of the divalent nature of the C6.5 diabody on its association with cell-bound HER2/*neu*, an in vitro cell-surface retention assay was performed. For this assay, the C6.5 scFv and diabody were biotinylated using an ImmunoPure NHS-LC-Biotinylation kit (no. 21430, Pierce, Rockford, IL, USA). Twelve micrograms of biotinylated C6.5 scFv or diabody were incubated with $1.2 \times 10^7$ HER2/*neu*-overexpressing SK-OV-3 (HBT 77; American Type Culture Collection, Rockville, MD, USA) cells (Weiner et al, 1993) in a total volume of 0.5 ml of FACS buffer (0.154 M sodium chloride, 10 mM sodium phosphate, 1% bovine serum albumin, 0.1% sodium azide, pH 7.2) for 30 min at room temperature. The cells were centrifuged at 500 ***g*** for 5 min at 4°C, washed with 10 ml of ice-cold FACS buffer two times and then resuspended gently in 12 ml of FACS buffer at 37°C. The cell suspensions were then incubated at 37°C with gentle shaking in a water bath. To decrease the rebinding of dissociated biotinylated diabody or scFv to the cells, at 15, 30, 45, 60, 90 and 120 min after commencing the incubation the suspensions were pelleted at 500 ***g***, the supernatants were aspirated and the cells were gently resuspended in fresh FACS buffer (37°C). Immediately after each round of pelleting and resuspension, 0.5-ml aliquots containing $5 \times 10^5$ cells were removed in triplicate (i.e. at 0, 15, 30, 45, 60, 90 and 120 min), placed on ice for 5 min and centrifuged at 500 ***g*** for 5 min at 4°C. After removing the supernatants from the aliquots, the cells were gently resuspended in 50 µl of ice-cold FACS buffer containing 50 µl of a 1:800 dilution of streptavidin-PE, incubated on ice for 30 min and washed twice with FACS buffer at 4°C. The cells were fixed with 1% paraformaldehyde and the degree of fluorescence was determined by analysis on a FACscan flow cytometer (Becton Dickinson, San Jose, CA, USA) as described (Weiner et al, 1993). $k_{off}$ was calculated assuming first-order kinetic using the formula $F_t = (F_i) \times (e^{-kt})$, where $F_t$ = fluorescence at time *t*, $F_i$ = fluorescence at time 0 and $k = k_{off}$. $t_{1/2}$ was calculated from $k_{off}$ using $F_t/F_i = 0.5$. Density of HER2/*neu* on the surface of SK-OV-3 cells was calculated to be 3.2–$4.8 \times 10^3$ molecules $\mu m^{-2}$, assuming a cell diameter of 10 µm and $1.0 \times 10^6$ HER2/*neu* ECD per cell (observations ranged from $1.0 \times 10^6$ to $1.5 \times 10^6$ in Scatchard assays; unpublished data).

 © Cancer Research Campaign 1998

**Figure 2** HPLC profiles of the C6.5 diabody and C6.5 scFv. After IMAC purification on a Ni++ agarose column, the C6.5 diabody and scFv were analysed on a Superdex 200 column (Pharmacia). The C6.5 scFv eluted from the Superdex 200 column as a single peak of approximately 25 kDa with minimal evidence of aggregation, while the diabody eluted as a single peak of approximately 50 kDa with no evidence of unassociated monomer

### Biodistribution studies

C6.5 diabody and scFv were radiolabelled with iodine-125 using the chloramine T (CT) method ($^{125}$I/scFv = 1:6; CT/scFv = 1:10) as previously described (Adams et al, 1992, 1993). The quality and immunoreactivity of the radiopharmaceuticals was evaluated by SDS-PAGE, high-performance liquid chromatography (HPLC) on a Superdex 75 column (Pharmacia) and in a live-cell binding assay as described (Adams et al, 1993). The immunoreactivities of the radiolabelled diabody and scFv monomer were found to be 87.6% and 65.3% respectively. The differences in immunoreactivity most likely reflect the prolonged association of the diabody with its antigen on the cell surface. Six- to eight-week-old CB.17 Icr *scid* mice were obtained from the Fox Chase Cancer Center Laboratory Animal Facility. Then, 2.5 × 10$^6$ human ovarian carcinoma SK-OV-3 cells were implanted s.c. on the abdomen of each mouse. When the tumours had achieved a size of 50–200 mg (approximately 8 weeks), Lugol's solution was placed in their drinking water to block thyroid accumulation of radioiodine, and biodistribution studies were initiated. Twenty micrograns (100 µl) of radioiodinated diabody or scFv were administered by i.v. tail vein injection to each mouse. Cohorts of five mice that had received the $^{125}$I-C6.5 diabody were sacrificed at 1, 4, 24, 48 and 72 h after injection and a single cohort of five mice that had received the $^{125}$I-C6.5 scFv monomer was sacrificed at 24 h after injection. The mean and s.e.m. of retention of each radiopharmaceutical in tissue (%ID g$^{-1}$) and blood (%ID ml$^{-1}$) was determined as described (Adams et al, 1993). Calculations of the estimated cumulative localization (AUC) of diabody in tissues and blood were determined using the NCOMP program (Laub, 1996). $t_{1/2}\alpha$ and $t_{1/2}\beta$ were calculated using the Rstrip program (Micromath, Salt Lake City, UT, USA). Significance levels were determined using a Student's $t$-test on the Statworks program (Cricket Software, Philadelphia, PA, USA).

### RESULTS

### Diabody expression and characterization

The C6.5 diabody and scFv were secreted from *E. coli* grown in shake flasks with typical yields of native protein after IMAC and HPLC purification of approximately 1.0 mg l$^{-1}$ for the diabody and 5 mg l$^{-1}$ for the scFv. The C6.5 scFv eluted from a Superdex 200 column as a single peak of approximately 25 kDa, with minimal

**Figure 3** In vitro characterization of the C6.5 diabody. C6.5 diabody and monomeric scFv were evaluated by surface plasmon resonance (BIAcore) as described in Methods. The association and dissociation kinetics are displayed for the diabody (●) and scFv monomer (○) forms of C6.5 (**A**). In vitro cell-surface retention profiles of biotinylated forms of the C6.5 diabody (●) and C6.5 monomer (○) were determined using SK-OV-3 cells, as described in the text, and the results are displayed; s.e.m.s are less than 2% of the value presented (**B**)

**Figure 4** The in vivo tumour targeting of radioiodinated C6.5 diabody was determined in a biodistribution study performed in SK-OV-3 tumour-bearing *scid* mice. The plotted values represent the mean tumour (●) and blood (○) retentions obtained from six mice per data point. The standard errors are indicated evidence of dimerization or aggregation (Figure 2A). The C6.5 diabody eluted from a Superdex 200 column as a single peak of approximately 50 kDa, with no evidence of unassociated monomer (Figure 2B). Both migrated under reducing conditions on 12% SDS-PAGE gels as single bands of approximately 27 kDa (data not shown).

The $K_d$ of the C6.5 diabody for HER2/*neu* ECD was determined by surface plasmon resonance in a BIAcore instrument to be 4.0 × $10^{-10}$ M ($k_{on}$ = 6.7 × $10^5$ $M^{-1}$ $s^{-1}$; $k_{off}$ = 2.7 × $10^{-4}$ $s^{-1}$) 40-fold lower than the $K_d$ of the C6.5 scFv (1.6 × $10^{-8}$ M; $k_{on}$ = 4.0 × $10^5$ $M^{-1}$ $s^{-1}$; $k_{off}$ = 6.3 × $10^{-3}$ $s^{-1}$) (Figure 3A). The decrease in $K_d$ was largely due to reduction in $k_{off}$, which correlated with a retention $t_{1/2}$ of 43 min, compared with 1.8 min for the scFv. In the BIAcore, the HER2/*neu* ECD is chemically coupled to a three dimensional matrix of carboxymethyl dextran, which bears little resemblance to the organization of HER2/*neu* on the cell surface. Therefore, the biological relevance of the increased affinity of the diabody was determined in an in vitro cell surface retention assay using biotinylated C6.5 diabody or scFv and human SK-OV-3 ovarian carcinoma cells overexpressing HER2/*neu*. In this assay, the quantity of biotinylated diabody or scFv retained on the surface of the SK-OV-3 cells over time was determined by flow cytometry. Significantly longer retention of the C6.5 diabody was observed compared with the C6.5 scFv ($t_{1/2}$ scFv = 2.5 min vs $t_{1/2}$ diabody = 5 h; $P < 0.001$) (Figure 2B). The results compare favourably to $t_{1/2}$ values calculated from the $k_{off}$ measured in the BIAcore of 1.8 min for the scFv and 43 min for the diabody. Thus the increase in apparent affinity was much greater on the cell surface than on the carboxymethyldextran surface of the BIAcore, despite the similarities in calculated density of HER2/*neu* sites (3.2–4.8 × $10^3$ sites $\mu m^{-2}$ on the cell surface vs 4.2 × $10^3$ sites $\mu m^{-2}$ on the sensor chip surface).

### Biodistribution assays

The in vivo targeting potential of the C6.5 diabody was assessed in *scid* mice bearing s.c. SK-OV-3 tumours overexpressing the HER2/*neu* antigen. The tumour, blood and organ retention of radioiodinated C6.5 diabody was determined at 1, 4, 24, 48 and 72 h after its i.v. administration. After the injections, the diabody displayed a rapid equilibration phase ($t_{1/2}\alpha$ = 0.67 h) and subsequent slower elimination phase ($t_{1/2}\beta$ = 6.42 h) from circulation, in a pattern characteristic of small scFv-based molecules (Figure 4). In contrast to the blood retention properties of the diabody, the quantity retained in tumour rose from 6.9% ID $g^{-1}$ at 1 hour post

**Table 1** Evaluation of [$^{125}$I]C6.5 diabody in tumour-bearing *scid* mice

| | C6.5 diabody | | | | | | | C6.5 scFv |
|---|---|---|---|---|---|---|---|---|
| | 1 h | 4 h | 24 h | 48 h | 72 h | Cumulative AUC | Tumour–organ AUC ratio | 24 h |
| Tumour[a] | 6.9 | 10.1 | 6.5 | 2.4 | 1.4 | 405 | – | 1.0 |
| Blood[b] | 21.5 (0.3) | 6.7 (1.5) | 0.7 (9.5) | 0.1 (22.5) | < 0.1 (24.0) | 133 | 3.0 | 0.1 (19.1) |
| Liver | 5.7 (1.2) | 2.8 (3.6) | 0.3 (21.0) | 0.1 (22.5) | 0.1 (15.0) | 137 | 3.0 | < 0.1 (25.4) |
| Kidneys | 16.9 (0.4) | 5.2 (1.9) | 1.1 (6.0) | 0.4 (6.7) | 0.3 (4.5) | 153 | 2.6 | 0.2 (6.3) |
| Lung | 17.0 (0.4) | 7.1 (1.4) | 0.7 (8.8) | 0.1 (19.7) | 0.1 (18.2) | 57 | 7.1 | 0.1 (17.0) |
| Spleen | 4.3 (1.6) | 3.5 (3.2) | 0.4 (16.1) | 0.1 (20.6) | 0.1 (17.0) | 61 | 6.6 | < 0.1 (24.5) |
| Heart | 13.1 (0.5) | 4.7 (2.2) | 0.4 (17.5) | 0.1 (31.0) | < 0.1 (31.7) | 93 | 4.4 | < 0.1 (37.2) |
| Stomach | 4.5 (1.6) | 7.9 (1.4) | 1.4 (5.7) | 0.3 (11.6) | 0.3 (6.6) | 129 | 3.1 | 0.2[c] (6.2) |
| Intestine | 1.6 (4.5) | 2.5 (4.6) | 0.3 (22.3) | 0.1 (33.4) | 0.1 (24.7) | 37 | 10.9 | < 0.1 (25.8) |
| Bone | 2.3 (3.3) | 1.9 (6.0) | 0.1 (40.6) | < 0.1 (84.3) | < 0.1 (65.0) | 31 | 13.1 | < 0.1[c] (34.2) |
| Muscle | 1.2 (5.8) | 1.5 (7.1) | 0.2 (34.3) | < 0.1 (44.6) | < 0.1 (53.4) | 37 | 10.9 | < 0.1 (49.8) |

[a]Expressed as %ID $g^{-1}$ tissue. [b]Expressed as %ID $ml^{-1}$ blood. [c]s.e.m. ≤45%. C.B 17/ICR-scid mice bearing 50- to 200-mg s.c. SK-OV-3 tumours were used in these studies. Cohorts of five mice per time point were given 20 µg of [$^{125}$I]C6.5 diabody by i.v. injection. The mice were sacrificed at the indicated times and the tumour, blood and normal tissue retention was determined and expressed as a percentage of the injected dose localized per g of tissue (%ID $g^{-1}$) or per ml of blood (%ID $ml^{-1}$) as described in Methods. Tumour–organ ratios are presented in parentheses. For each value presented, the s.e.m. was less than 30%, unless otherwise indicated. The cumulative diabody retention (AUC) in each tissue was determined as described and is expressed in arbitrary units to facilitate the determination of the tumour to organ AUC ratios.

© Cancer Research Campaign 1998

injection to a peak of 10.1 %ID g$^{-1}$ at 4 h post injection and slowly decreased to 6.5% ID g$^{-1}$ and 1.4% ID g$^{-1}$ at 24 and 72 h respectively (Table 1 and Figure 4). The retention of the diabody in normal organs reflected the concentration present in blood over the course of the study with the notable exception of the kidneys, which function as the major elimination route for scFv-based reagents (Table 1). The cumulative residence of the radioiodinated diabody in tumour and normal organs, expressed as AUCs, was determined to predict the therapeutic potential for this molecule. Over the course of the study, favourable tumour to organ AUC ratios were observed for a number of organs, including liver (3.0), spleen (6.6), bone (13.1), kidneys (2.6) and blood (3:1) (Table 1). While the activity in the bone marrow compartment is difficult to measure directly, it is routinely estimated based upon the observation that one-fourth of the bone marrow compartment is composed of blood (Siegel et al, 1990). As HER2/*neu* is not expressed on cells in the marrow, the diabody will not specifically bind to marrow, just as it does not bind to other tissues lacking HER2/*neu* (e.g. liver, spleen and muscle). Therefore, the radioiodinated diabody present in the bone marrow compartment can be solely attributed to that present in the blood portion of the bone marrow. Accordingly, the tumour to bone marrow ratio was estimated as 12:1 (25% of the tumour–blood ratio).

The biodistribution of the [$^{125}$I]C6.5 scFv monomer was performed at 24 h after administration for comparative purposes and was found to be virtually identical to that previously reported for this and other scFv monomers of similar affinity, with 1.0 % ID g$^{-1}$ retained in tumour, 0.04 % ID g$^{-1}$ in liver and 0.05 % ID ml$^{-1}$ in blood (Table 1) (Colcher et al, 1990; Milenic et al, 1991; Adams et al, 1993; Schier et al, 1995). This clearly demonstrated the significantly increased tumour retention ($P = 0.00043$) conferred by the diabody format. The prolonged blood retention of the larger diabody molecule may also account for some of the increased tumour retention. This is evidenced by the 24-h tumour–blood ratios of about 9:1 for the diabody and 20:1 for the monomer.

### DISCUSSION

Here we describe the production and in vitro and in vivo properties of the C6.5 diabody molecule specific for HER2/*neu*. The C6.5 diabody was expressed and purified in high yield from *E. coli* as native protein without refolding. Compared with the scFv from which it was derived, the diabody exhibited a significantly lower $K_d$ and slower $k_{off}$ from HER2/*neu* that was either immobilized on a BIAcore sensor chip or as expressed on the surface of tumour cells. In vivo, radioiodinated C6.5 diabody displayed an excellent balance of quantitative tumour deposition and specificity. Peak tumour values of 10 %ID g$^{-1}$ were observed at 4 h after intravenous administration and persisted through 24 h (6.5 %ID g$^{-1}$) and 72 h (1.2 %ID g$^{-1}$) post injection. In contrast, the diabody was rapidly cleared from the circulation and antigen-negative organs, as its molecular weight (50 kDa) is less than the renal threshold. As a result, significantly more diabody was retained in tumour than in any other organ at all but the earliest time points studied. This yielded tumour–normal organ AUCs of 3:0 (tumor–blood) to 13.1:1 (tumour–bone). Furthermore, as we and others have previously demonstrated, antibody-based molecules with sizes beneath the renal threshold for first-pass clearance are typically eliminated in a biphasic manner, with a rapid initial equilibration phase and a slower elimination phase (reviewed in Huston et al, 1996). This suggests that the sampling times used in this study may have exaggerated the blood AUC value for the interval between 4 and 24 h. Thus, it is likely that the inclusion of additional sampling times (i.e. 6, 12 and 16 h post injection) would reveal lower blood retentions and hence, more specific tumour localization. As the C6.5 diabody was developed from a phage display-derived scFv, a C6.5 IgG molecule was not available for a direct comparison between these two divalent structures. However, a reasonable comparison can be made using an IgG molecule specific for a different epitope on the HER2/*neu* antigen. We have previously reported on the distribution of 741F8 IgG, which, like many other monoclonal antibodies targeting cell-surface tumour-associated antigens, exhibits a high degree of tumour uptake (e.g. 20 %ID g$^{-1}$) with very poor targeting specificity (tumour–blood ratios ≤ 1:1) (Weiner et al, 1995). Therefore, even though the degree of tumour retention observed with the C6.5 diabody was less than that observed with anti-HER2/*neu* IgG, the increased targeting specificity associated with the diabody format results in an advantage.

Compared with the scFv, the increased tumour deposition of the diabody could result from its increased size or increase in apparent affinity (avidity). The increased size of the C6.5 diabody led to a slower redistribution and elimination $t_{1/2}$ than was observed for the C6.5 scFv. This leads to higher blood levels and prolongation of the concentration gradient for diffusion from blood into tumour. However, Fab are of similar size and have similar pharmacokinetics, but do not provide as great an increment in quantitative tumour retention compared with scFv (Milenic et al, 1991; Adams et al, 1993). Thus, size alone is unlikely to account for the increased tumour deposition and retention of the diabody, which instead must be at least partly due to an increase in apparent affinity resulting from avidity. A priori, it was unclear to what extent an increase in apparent affinity would occur with the divalent diabody molecule. The Fab arms of the IgG molecule are extremely flexible, because of the hinge (Ferencik, 1993). In contrast, the two binding heads of the diabody are oriented 180° apart in a rigid configuration (Perisic et al, 1994). Thus, the extent to which the diabody could engage two antigens simultaneously, particularly on the cell surface, was unclear. As determined using surface plasmon resonance in a BIAcore, the apparent affinity of the diabody is 40-fold higher than the scFv, largely because of a 40-fold reduction in $k_{off}$. The dissociation of diabody from the cell surface was sevenfold slower than observed on the BIAcore, with a $k_{off}$ approximately 280-fold slower than the scFv. As the calculated antigen density on the BIAcore sensor chip surface and the cell surface are approximately the same, these differences may result from the greater mobility of HER2/*neu* ECD in the cell membrane, leading to bivalent binding without steric strain.

Monoclonal antibody (MAb)-based radioimmunotherapy (RAIT) has shown notable promise in the treatment of haematological malignancies (Kaminski et al, 1993; Press et al, 1993), but progress in the therapy of solid tumours has been hindered by a number of factors dictated by tumour physiology (Jain, 1990). First, the disordered vasculature of solid tumours leads to a heterogeneous intratumoral distribution of MAb. Second, the paucity of draining lymphatics in tumours results in elevated hydrostatic pressure, limiting the diffusion of large molecules such as IgG to 100 μm in 1 h, 1 mm in about 2 days and 1 cm in about 7–8 months. To obtain sufficient tumour localization for radiolabelled MAb to provide therapeutic effects, the MAb must remain in circulation long enough to diffuse from blood into tumour. At the same time, the radiolabelled MAb must be eliminated from circulation rapidly enough to diminish normal organ retention and prevent unacceptable toxicities.

© Cancer Research Campaign 1998

To achieve successful RAIT, a proper balance must be established between these competing requirements. We hypothesized that an appropriate balance could be accomplished by using small, high-affinity, multivalent, antibody-based molecules. Decreasing the size of the molecule increases both its diffusion rate into tumour (Jain, 1990) and its rate of elimination from circulation, thus enhancing both the degree of tumour penetration and the specificity of tumour retention. While the optimal size for an antibody-based construct has yet to be identified, we believe it will fall below the renal threshold for first-pass clearance (about 65 kDa). When administered by a continuous i.v. infusion, such molecules could be maintained at steady-state levels in circulation, and controlled gradients from blood into tumour could be established. This would facilitate deep penetration into tumour and highly specific tumour retention when the molecules are rapidly eliminated from circulation upon the termination of the infusion. A variety of molecular structures that span a wide range of sizes are available. These include 80-kDa $(scFv\text{-}CH3)_2$ minibodies (Hu Shizhen et al, 1996), 50-kDa diabodies (Holliger et al, 1993), 27-kDa scFv (Bird et al, 1988; Huston et al, 1988) and individual 12- to 13-kDa $V_H$ or $V_L$ chains (Ward et al, 1989). While the smallest molecules will be capable of the greatest diffusion into solid tumours, their administration will require careful management to maintain the blood concentrations required to permit diffusion through tumour. Increasing the functional affinity may help 'trap' the scFv that diffuses into tumour, localizing it long enough to facilitate therapeutic applications. This can be accomplished by manipulating the intrinsic affinity properties (Schier et al, 1996) or through the creation of multivalent binding proteins. While Weinstein has hypothesized that the diffusion of high-affinity MAb into tumour is hindered by binding to antigen-bearing cells close to blood vessels (Fujimori et al, 1989; Juweid et al, 1992), this may be overcome by enhancing the diffusion gradient from blood into tumour through the administration of large doses. Finally, the potential of engineered antibody-based proteins to target tumours in humans in a highly specific manner was recently demonstrated using radioimmunoimaging performed by Begent et al (1996). Given that successful tumour localization in the above study was achieved with a small, monovalant scFv, it is our belief that the larger, divalant diabody molecule used here will also exhibit impressive tumour targeting in patients.

Of the divalent scFv-based molecules produced to date, before this study, reports of in vivo assays only exist for the $(scFv')_2$ and the minibody. Previously, we have shown that the tumour retention of a 20-μg dose of the anti-HER2/*neu* 741F8 $(scFv')_2$ in *scid* mice bearing relevant tumours is twice that seen with 741F8 scFv monomer (Adams et al, 1993). While the specificity of tumour retention at 24 h post injection was very high, as evidenced by tumour–blood and tumour–muscle ratios of 10:1 and 75:1, respectively, the quantity of $(scFv')_2$ retained in tumour (1.6 %ID $g^{-1}$) was insufficient to mediate therapeutic effects or predict for therapeutic dosimetry in tumours. Hu Shi-Zhen et al (1996) have recently reported excellent selective tumour retention after the administration of small quantities (0.1–0.2 μg per mouse, or 0.005–0.01 μg/$g^{-1}$ body weight) of anti-CEA $(scFv\text{-}C_H3)_2$ minibodies to tumour-bearing athymic mice, with average 24-h retentions of 29 %ID $g^{-1}$ and 8 %ID $g^{-1}$ in tumour with flex and LD minibodies respectively. While the mass of the C6.5 diabody (50 kDa) lies just below the renal threshold for first-pass clearance, the two minibody species have molecular weights of approximately 80 kDa and are above the threshold. This is evidenced by the faster clearance of the diabody from circulation (0.7 %ID $ml^{-1}$ vs 2.1 %ID ml,$^{-1}$ respectively, for the diabody and minibodies at 24 h post injection), which probably leads to a lower cumulative blood, and hence marrow, exposure for the diabody. However, the greater peak tumour retention of the minibody leads to similar tumour–blood AUCs for both molecules. Clearly, the parallel evaluation of identical doses of a series of reagents (i.e. scFv, $(scFv')_2$, diabody and minibody) with identical specificity is desirable to definitively address the role of size on the in vivo tumour-targeting properties of these recombinant antibody-based molecules.

The cumulative retention (AUC) of C6.5 diabody in tumour and normal tissues was calculated to predict the therapeutic potential of diabodies as vehicles for RAIT. RAIT efficacy is dependent upon the delivery of lethal doses of radiation to tumour without exceeding the doses tolerated by the bone marrow (200–300 cGy) and organs involved in the catabolism of the radiopharmaceutical, such as the kidneys (1500 cGy) and the liver (4000 cGy) (Bentel et al, 1989). In this study with the C6.5 diabody, we calculated tumour to organ ratios ranging from 3:1 to 13:1. The tumour–bone marrow estimate of 12:1 and tumour–kidney value of 2.6:1 would permit the delivery of approximately 4000 cGy to tumour at a marrow dose of 250 cGy. This represents a significant improvement over results in preclinical models observed with other antibody-based molecules, including scFv (Adams et al, unpublished results), $(scFv')_2$ (Weiner et al, 1995), Fab (Yorke et al, 1991), $F(ab')_2$ (Stein et al, 1991, 1994) and IgG (Stein et al, 1991; Molthoff et al, 1991, 1992). While the predicted tumour–blood AUCs for Flex minibodies are similar to those reported here for the C6.5 diabody, the smaller diabody structure may confer an advantage when penetration of large solid tumours is required.

As the divalent binding of a diabody molecule to antigen on the surface of a tumour cell molecule is dependent upon both the density of the antigen and its orientation, it is likely that such binding would only occur when the antigen density is high. While the C6.5 diabody remains bound to the tumour cells in vitro (Figure 3B) and is retained in tumour in vivo (Figure 4) significantly longer than is its monomeric scFv form, it is likely that diabody bound to normal tissue expressing low concentrations of HER2/*neu* would bind in a monovalent manner and exhibit the rapid dissociation kinetics characteristic of the C6.5 scFv. To confirm this hypothesis, the in vitro and in vivo binding profiles of C6.5 diabody and C6.5 scFv require evaluation in tumour cell lines and tumours with a wide range of HER2/*neu* expression.

If the prolonged retention of the C6.5 diabody on the surface of cells and in tumours overexpressing HER2/*neu* is mediated by divalent binding, it may exert a direct biological impact on these cells. Homodimerization of HER2/*neu* or heterodimerization of HER2/*neu* with c-erbB-3 or c-erbB-4 has recently been found to be required for signal transduction after the binding of heregulin to c-erbB-3 or c-erbB-4 (Earp et al, 1995; Wallasch et al, 1995). The possibility that divalent binding of two HER2/*neu* molecules by C6.5 diabody facilitates the homodimerization of HER2/*neu* with subsequent signal transduction is intriguing. Alternatively, it is possible that cytostatic effects could be triggered by the immobilization of HER2/*neu* on the cell surface to prevent the homodimerization of the molecule's transmembrane region. Either of these mechanisms may be responsible for reports of synergistic effects between some anti-HER2/*neu* monoclonal antibodies and chemotherapeutic agents, such as taxol or cisplatin (Hancock et al, 1991). Accordingly, the potential of the C6.5 diabody to dimerize HER2/*neu*, trigger signal transduction and inhibit tumour cell

© Cancer Research Campaign 1998

growth in the presence of chemotherapeutic agents has been studied. However, when the C6.5 diabody has been assayed for growth inhibition potential by in vitro MTT [3-(4,5-dimethylthiazol-2-yl)-2,5-diphenyltetrazolium bromide] incorporation assays, concentrations of up to 10 μg $ml^{-1}$ for 7 days do not significantly inhibit the growth of SK-OV-3 cells overexpressing the HER2/*neu* antigen (unpublished results). Accordingly, it is probable that the C6.5 diabody by itself is not capable of exerting cytostatic effects.

Continued improvements in antibody engineering have led to increasingly sophisticated structures that address impediments to successful tumour targeting. The C6.5 diabody may be an effective targeting vehicle for RAIT. In addition, this molecule may provide a useful platform for the creation of affinity mutants with slower $k_{off}$ rates or for the creation of fusion proteins containing other antibodies, cytokines, chemotactic factors or toxins.

### ACKNOWLEDGEMENTS

This study was supported by National Cancer Institute (NCI) grant CA 65559, Department of Defense (DOD) grant DAMD17-94-J-4433, NCI grant CA06927, an appropriation from the Commonwealth of Pennsylvania, the Bernard A and Rebecca S Bernard Foundation, the Frank Strick Foundation and the CaPCURE Foundation. The authors would like to thank Heidi Simmons, Eva Horak, Anne Amoroso and James Babb of the Fox Chase Cancer Center and Cindy Wong, Michael Yim, Donnie Tran and Grete Hemmingder of the University of California, San Francisco, for their expert technical assistance.

### REFERENCES

Adams GP, DeNardo SJ, Amin A, Kroger LA, DeNardo GL, Hellström I and Hellström KE (1992) Comparison of the pharmacokinetics in mice and the biological activity of murine L6 and human-mouse chimeric Ch-L6 antibody. *Antibody Immunoconj Radiopharmaceut* **5**: 81–95

Adams GP, McCartney JE, Tai M-S, Oppermann H, Huston JS, Stafford WF, Bookman MA, Fand I, Houston LL and Weiner LM (1993) Highly specific *in vivo* tumour targeting by monovalent and divalent forms of 741F8 anti-c-erbB-2 single-chain Fv. *Cancer Res* **53**: 4026–4034

Adams GP, McCartney JE, Wolf EJ, Tai M-S, Schier R, Stafford WF, Marks JD, Bookman MA, Huston JS and Weiner LM (1996). Influence of avidity on the tumor retention of monospecific and bispecific anti-c-erbB-2 single-chain Fv dimers. *Proc Am Assoc Cancer Res* **37**: 472

Allred DC, Clark GM, Molina R, Tandon AK, Schnitt SJ, Gilchrist KW, Osborne CK, Tormey DC and McGuire WL (1992) Overexpression of HER-2/*neu* and its relationship with other prognostic factors change during the progression of in situ to invasive breast cancer. *Hum Pathol* **23**: 974–979

Beaumier PL, Krohn KA, Carrasquillo JA, Eary J, Hellström I, Hellström KE, Nelp WB and Larson SM (1985) Melanoma localization in nude mice with monoclonal Fab against p97. *J Nucl Med* **26**: 1172–1179

Begent RH, Verhaar MJ, Chester KA, Casey JL, Green AJ, Napier MP, Hope-Stone LD, Cushen N, Keep PA, Johnson CJ, Hawkins RE, Hilson AJ and Robson L (1996) Clinical evidence of efficient tumor targeting based upon single-chain Fv antibody selected from a combinatorial library. *Nature Med* **2**: 979–984

Bentel GC, Nelson CE and Noell KT (1989) *Treatment Planning and Dose Calculation in Radiation Oncology*. Pergamon Press: New York

Berchuck A, Kamel A, Whitaker R, Kerns B, Olt G, Kinney R, Soper JT, Dodge R, Clarke-Pearson DL and Marks P (1990) Overexpression of HER2/*neu* is associated with poor survival in advanced epithelial ovarian cancer. *Cancer Res* **50**: 4087–4091

Bird RE, Hardman KD, Jacobson JW, Johnson S, Kaufman BM, Lee S-M, Lee T, Pope SH, Riordan GS and Whitlow M (1988) Single-chain antigen-binding proteins. *Science* **242**: 423–426

Breitling SD, Seehaus T, Klewinghaus I and Little M (1991) A surface expression vector for antibody screening. *Gene* **104**: 147–153

Colcher DR, Bird R, Roselli M, Hardman KD, Johnson S, Pope S, Dodd SW, Pantoliano MW, Milenic DE and Schlom J (1990) In vivo tumor targeting of a recombinant single-chain antigen-binding protein. *J Natl Cancer Inst* **82**: 1191–1197

Earp HS, Dawson TL, Li X and Yu H (1995) Heterodimerization and functional interaction between EGF receptor family members: a new signaling paradigm with implications for breast cancer research. *Breast Cancer Res Treat* **35**: 115–132

Ferencik M (1993) The Immunoglobulins. In *Handbook of Immunology*, pp. 69–109. Chapman & Hall: London

Fujimori K, Covell DG, Fletcher JE and Weinstein JN (1989) Modeling analysis of the global and microscopic distribution of immunoglobulin G, F(ab′)2 and Fab in tumors. *Cancer Res* **49**: 5656–5663

Hancock MC, Langton BC, Chan T, Toy P, Monahan JJ, Mischak RP and Shawver LK (1991) A monoclonal antibody against the c-*erbB*-2 protein enhances the cytotoxicity of *cis*-Diamminedichlorplatinum against human breast and ovarian tumor cell lines. *Cancer Res* **51**: 4575–4580

Hochuli E, Bannwarth W, Dobeli H, Gentz R and Stuber D (1988) Genetic approach to facilitate purification of recombinant proteins with a novel metal chelate. *Bio/Technology* **6**: 1321–1325

Holliger P, Prospero T and Winter G (1993) 'Diabodies': small bivalent and bispecific antibody fragments. *Proc Natl Acad Sci USA* **90**: 6444–6448

Hu Shi-zhen SL, Raubitschek A, Sherman M, Williams LE, Wong JYC, Shively JE and Wu AM (1996) Minibody: a novel engineered anti-carcinoembryonic antigen antibody fragment (single-chain Fv-CH3) which exhibits rapid, high-level targeting of xenografts. *Cancer Res* **56**: 3055–3061

Huston JS, Levinson D, Mudgett-Hunter M, Tai M-S and Novotny J (1988) Protein engineering of antibody binding sites: recovery of specific activity in an antidigoxin single-chain Fv analogue produced in *E coli*. *Proc Natl Acad Sci USA* **85**: 5879–5883

Huston JS, George AJT, Adams GP, Stafford WF, Jamar F, Tai M-S, McCartney JE, Oppermann H, Heelan BT, Peters AM, Houston LL, Bookman MA, Wolf EJ and Weiner LM (1996) Single-chain Fv radioimmunotargeting. *Q J Nucl Med* **40**: 320–333

Jain RK (1990) Physiological barriers to delivery of monoclonal antibodies and other macromolecules in tumors. *Cancer Res* **50**: (suppl.) 814s–819s

Jønsson U, Faegerstam L, Ivarsson B, Lundh K, Løfås S, Persson B, Roos H, Rønnberg I, Sjølander S, Stenberg E, Ståhlberg R, Urbaniczky C, Østlin H and Malmqvist M (1991) Real-time biospecific interaction analysis using surface plasmon resonance and a sensor chip technology. *Bio Techniques* **11**: 620–627

Juweid MN, R Paik, Perez-Bacete C, M Sato, J van Osdol W and Weinstein JN (1992) Micropharmacology of monoclonal antibodies in solid tumors: direct experimental evidence for binding site barrier. *Cancer Res* **52**: 5144–5153

Kaminski MS, Zasady KR, Francis IR, Milik AW, Ross CW, Moon SD, Crawford SM, Burgess JM, Petry NA, Butchko GM, Glenn SD and Wahl RL (1993) Radioimmunotherapy of B-cell lymphoma with 131I-anti-B1 (anti-CD20) antibody. *New Engl J Med* **329**: 459–465

Karlson RA, Michaelsson A and Mattsson L (1991) Kinetic analysis of monoclonal antibody-antigen interactions with a new biosensor based analytical system. *J Immunol Methodol* **145**: 229–240

King CR, Kraus MH and Aaronson SA (1985) Amplification of a novel v-*erbB*-related gene in a human mammary carcinoma. *Science* **229**: 974–976

Kraus MH, Popeseu NC, Amsbaugh SC and King CR (1987) Overexpression of the EGF receptor-related proto-oncogene *erbB*-2 in human mammary tumor cell lines by different molecular mechanisms. *EMBO J* **6**: 605–610

Laub P and Gallo J (1996) NCOMP – a windows-based computer program for noncompartmental analysis of pharmacokinetic data. *J Pharmaceut Sci* **85**: 393–395

Marks JD, Hoogenboom HR, Bonnert TP, McCafferty J, Griffiths AD and Winter G (1991) By-passing immunization. Human antibodies from V-gene libraries displayed on phage. *J Mol Biol* **222**: 581–597

McCartney JE, Tai M-S, Hudziak RM, Adams GP, Weiner LM, Jin DJ, Stafford III WF, Liu S, Bookman MA, Laminet AA, Fand I, Houston LL, Oppermann H and Huston JS (1994). Engineering disulfide-linked single-chain Fv dimers [(sFv′)2] with improved solution and targeting properties: anti-digoxin 26–10 (sFv′)2 and anti-c-erbB-2 741F8 (sFv′)2 made by protein folding and bonded through C-terminal cysteinyl peptides. *Protein Engineering* **18**: 301–314

Milenic DE, Yokota T, Filpula DR, Finkelman MAJ, Dodd SW, Wood JF, Whitlow M, Snoy P and Schlom J (1991) Construction, binding properties, metabolism, and targeting of a single-chain Fv derived from the pancarcinoma monoclonal antibody CC49. *Cancer Res* **51**: 6363–6371

Molthoff CFM, Pinedo HM, Schlupper HMM, Nijman HW and Boven E (1992) Comparison of the pharmacokinetics, biodistribution and dosimetry of monoclonal antibody OC125, OV-TL3 and 139H2 as IgG and F(ab′)2 fragments in experimental ovarian cancer. *Br J Cancer* **65**: 677–683

© Cancer Research Campaign 1998

Pack PK, M Schroeckh V, Knüpfer U, Wenderoth R, Riesenberg D and Plückthun A (1993) Improved bivalent miniantibodies, with identical avidity as whole antibodies, produced by high cell density fermentation of *Escherichia coli*. BIO/Technology **11**: 1271–1277

Pack PPA (1992) Miniantibodies: use of amphipathic helices to produce functional, flexably linked dimeric Fv fragments with high avidity in *Escherichia coli*. *Biochemistry* **31**: 1579–1584

Perisic O, Webb PA, Hollinger P, Winter G and Williams RL (1994) Crystal structure of a diabody, a bivalent antibody fragment. *Structure* **2**: 1217–1226

Press OW, Eary JF, Appelbaum FR, Martin PJ, Badger CC, Nelp WB, Glenn S, Butchko G, Fisher D, Porter B, Matthews DC, Fischer LD and Bernstein ID (1993) Radiolabeled-antibody therapy of B-cell lymphoma with autologous bone marrow support. *New Engl J Med* **329**: 1219–1224

Schier R, Marks JD, Wolf E, Apell G, Wong C, McCartney J, Bookman M, Huston J, Weiner L and Adams GP (1995) In vitro and in vivo characterization of a human anti-c-erbB2 single chain Fv isolated from a filamentous phage antibody library. *Immunotechnology* **1**: 73–81

Schier R, McCall A, Adams GP, Marshall KW, Merritt H, Yim M, Crawford RS, Weiner LM, Marks C and Marks JD (1996) Isolation of picomolar affinity anti-c-erbB-2 single-chain Fv by molecular evolution of the complementarity determining regions in the center of the antibody binding site. *J Mol Biol* **263**: 551–567

Siegel JA, Wessels BW, Watson EE, Stobin MG, Vriesendorp HM, Bradley EW, Badger CC, Brill AB, Kwok CS, Stickney DR, Eckerman KF, Fisher DR, Buchsbaum DJ and Order SE (1990) Bone marrow dosimetry and toxicity for radioimmunotherapy. *Antibody Immunoconj Radiopharmaceut* **3**: 213–233

Slamon DJ, Clark GM, Wong SG, Levin WJ, Ullrich A and McGuire WL (1987) Human breast cancer: correlation of relapse and survival with amplification of the HER-2/*neu* oncogene. *Science* **235**: 177–182

Stein R, Chen S, Sharkey RM and Goldenberg DM (1991) Radioimmunotherapy of human non-small cell carcinoma of the lung xenografts with $^{131}$I-labeled monoclonal antibody RS7-3G11. *Antibody Immunoconj Immunopharmaceut* **4**: 703–712

Stein R, Blumenthal R, Sharkey RM and Goldenberg DM (1994) Comparative biodistribution and radioimmunotherapy of monoclonal antibody RS7 and its F(ab′)2 in nude mice bearing human tumor xenografts. *Cancer* **73**: 816–823

van de Vijver M, van de Bersselaar R, Devilee P, Cornelisse C, Peterse J and Nusse R (1987) Amplification of the *neu* (*c-erbB*-2) oncogene in human mammary tumors is relatively frequent and is often accompanied by amplification of the linked c-erbA oncogene. *Mol Cell Biol* **7**: 2019–2023

Wallasch C, Weiss FU, Niederfellner G, Jallal B, Issing W and Ullrich, A (1995) Heregulin-dependent regulation of HER2./neu oncogenic signaling by heterodimerization with HER3. *EMBO J* **14**: 4267–4275

Ward ES, Gussow D, Griffiths AD, Jones PT and Winter G (1989) Binding activities of a repertoire of single immunoglobulin variable domains secreted from *Escherichia coli. Nature* **341**: 544–546

Weiner LM, Holmes M, Richeson A, Godwin A, Adams GP, Hsieh-Ma ST, Ring DB and Alpaugh RK (1993) Binding and cytotoxicity characteristics of the bispecific murine monoclonal antibody 2B1. *J Immunol* **151**: 2877–2887

Weiner LM, Houston LL, Huston JS, McCartney JE, Tai M-S, Apell G, Stafford WF, Bookman MA, Gallo JM and Adams GP (1995) Improving the tumor-selective delivery of single-chain Fv molecules. *Tumor Targeting* **1**: 51–60

Wolf EA, Schreiber GJ, Cosand WL and Raff HV (1993) Monoclonal antibody homodimers: enhanced antitumor activity in nude mice. *Cancer Res* **53**: 2560–2565

Yokota T, Yamamoto T, Miyajima N, Toyoshima K, Nomura N, Sakamoto H, Yoshida T, Terada M and Sugimura T (1988) Genetic alterations of the *c-erb*B2 oncogene occur frequently in tubular adenocarcinoma of the stomach and are often accompanied by amplification of the *v-erb*A homologue. *Oncogene* **2**: 283–287

Yorke ED, Beaumier PL, Wessels BW, Fritzberg AR and Morgan C (1991) Optimal antibody-radionuclide combinations for clinical radioimmunotherapy: a predictive model based upon mouse pharmacokinetics. *Nucl Med Biol Int J Radiat Appl Instrum* Part B **18**: 827–835

© Cancer Research Campaign 1998